# Spatio-temporal coordination among functional residues in protein

**DOI:** 10.1038/srep40439

**Published:** 2017-01-16

**Authors:** Sutapa Dutta, Mahua Ghosh, J. Chakrabarti

**Affiliations:** 1 Department of Chemical, Biological and Macro-Molecular Sciences, S. N. Bose National Centre for Basic Sciences, Sector III, Block JD, Salt Lake, Kolkata 700106, India; 2 Also at Unit of Nanoscience and Technology-II and The Thematic Unit of Excellence on Computational Materials Science, S. N. Bose National Centre for Basic Sciences, Sector III, Block JD, Salt Lake, Kolkata 700106, India

## Abstract

The microscopic basis of communication among the functional sites in bio-macromolecules is a fundamental challenge in uncovering their functions. We study the communication through temporal cross-correlation among the binding sites. We illustrate via Molecular Dynamics simulations the properties of the temporal cross-correlation between the dihedrals of a small protein, ubiquitin which participates in protein degradation in eukaryotes. We show that the dihedral angles of the residues possess non-trivial temporal cross-correlations with asymmetry with respect to exchange of the dihedrals, having peaks at low frequencies with time scales in nano-seconds and an algebraic tail with a universal exponent for large frequencies. We show the existence of path for temporally correlated degrees of freedom among the functional residues. We explain the qualitative features of the cross-correlations through a general mathematical model. The generality of our analysis suggests that temporal cross-correlation functions may provide convenient theoretical framework to understand bio-molecular functions on microscopic basis.

Quite often bio-macromolecules undergo cascade of ligand bindings at different sites. Such binding events not only control cellular processes, but also lie at the heart of technological applications with bio-molecules as scaffold^1^. The microscopic basis of communication among the binding sites in bio-macromolecules is one of the fundamental questions which have drawn considerable attention, but still remains largely obscure. Motivated by this, we make attempt here to understand communication among functional residues in proteins, incorporating information of microscopic motions.

We consider the case of a small protein, ubiquitin^2^ (Ub) (PDB id: 1UBQ, see Supplementary Fig. S1(a)) involved in ubiquitination^3^,^4^, a process ubiquitous among the eukaryotes by which ubiquitin attaches with a target protein to degrade the latter. The process is initiated by covalent attachment of Adenosine mono-phosphate (AMP) to C-terminal Glycine, G76 of Ub. Following this, ubiquitin activation enzyme-E1 binds at different residues of Ub^3^,^4^,^5^,^6^, (see Supplementary Figs S1(b)–(d)). The question is: How do the spatially distant residues get temporally correlated so that the binding information at one site at a given time affects the binding at other sites at a later time?

Although experimental probes are limited^7^,^8^, recent simulation works in this direction emphasize on the covariance (Pearson Correlation Coefficient) between the instantaneous values of microscopic degrees of freedom, like the angles between atomic planes known as dihedral angles in a protein^9^,^10^,^11^,^12^,^13^,^14^,^15^. Non-zero but very small values of Pearson Correlation Coefficient have been observed among the dihedral angles of functional but spatially distant residues in Ub^9^. However, information provided by Pearson Correlations is far from complete. Macromolecular binding takes place typically by rotational diffusion ranging in timescales of tens of nano-seconds (ns)^16^, so that the binding surfaces are mutually exposed. This means that the changes at sites upon first ligand binding must affect the downstream binding sites till this time. Such temporal information are absent in Pearson Correlation Coefficient. The information entropy transfer^15^,^17^ approaches has been proposed to causally connect residues depending upon the history of their correlated fluctuations which includes all non-linear coupling between the fluctuating variables. Not only that the computation of information entropy transfer is quite involved, but also the physical mechanisms leading to the non-linear couplings is not understood, primarily due to lack of experimental probes. Moreover, time is introduced in the formalism on an ad-hoc basis^17^.

Alternatively, temporal cross-correlations of fluctuations of two physical quantities *A(t*) and *B(t*′) at times *t* and *t*′ with respect to their mean values, also known as two-point correlations functions^18^,^19^, are used to describe time scales of correlated stochastic processes^20^. The equal time correlation function, *t* = *t*′, is the statistical Pearson Correlation. Temporal cross-correlation functions can be thought of generalization of Pearson correlation in time domain. Two-point correlation functions do not contain information on non-linear coupling. One major advantage of two-point correlation functions is that they are experimentally accessible by scattering techniques. Interestingly, temporal cross-correlations between fluorescence intensities show asymmetry with inversion in time which has been used to study co-localization of proteins^21^. This observation seems to suggest that temporally causal connections can be extracted from the two-point correlation functions. The power of two-point cross-correlation functions has not been exploited for in-depth understanding of bio-molecular phenomena. Time dependent dihedral cross-correlation functions (TDCF) have been employed for correlation between protein residues only up to a few hundred pico-seconds (ps)^22^, far too low compared to the bio-molecular binding time scales to have functional relevance. We have established in an earlier work^23^ from much longer simulations that TDCF can relate large scale changes in a protein upon ligand binding.

With this backdrop we examine here the TDCF by long computer simulations and mathematical modeling to understand functional co-ordination among residues. We show that the TDCF can explain the causal connection between the functional residues of Ub in the time scale of tens of nano-seconds. We explain the qualitative features of TDCF using simple mathematical model. Interesting aspect of our result is that TDCFs contain temporal information which may be useful to understand biological processes which are orders of magnitude slower than atomic motions without invoking the non-linear effects.

## Molecular Dynamics and TDCF

**Figure 1:**
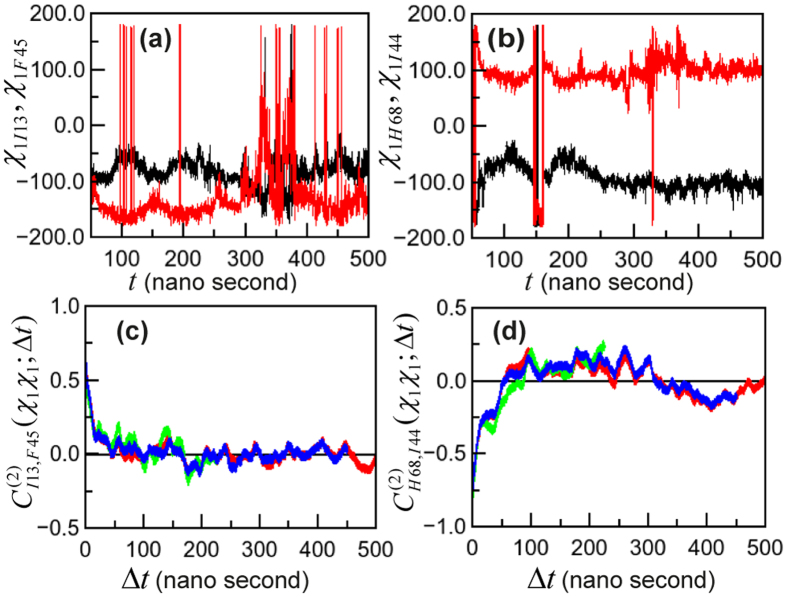
(**a**) Dihedral angles as functions of time *t*; 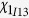 (black) and 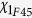 (red). (**b**) 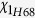 (black) and 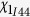 (red). (**c**) Convergence of TDCFs for three different *t* = 500 ns (green), 950 ns (blue) and 1.05 μs (red); 
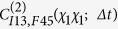
. (**d**) 
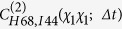
 as a function of *Δt*.

We perform 1.05 microseconds (μs) long all-atom MD simulations^24^ (see Methods, *Note 1*) for Ub with initial input from crystal structure^2^ in explicit water. We analyze data using the portion of the simulated trajectory where the root mean squared deviation (RMSD) of the backbone atoms is saturated (excluding initial 50 ns, see Supplementary Fig. S2). We calculate the dihedral angles for backbone (*φ, ψ*) and side-chain (*χ*_1_) of the residues of the protein. We plot the dihedrals *χ*_1_ for residue pairs Isoleucine: I13 and Phenylalanine: F45 denoted by 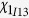 and 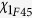 respectively as functions of time in Fig. 1(a). I13 and F45 have backbone distance (*d*_*α*−*α*_), given by that of their *C*_*α*_ atoms as large as 1.5 nm. Despite that both 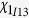 and 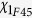 exhibit correlated behavior: The increase in one is coupled to increase in the other till very long time. Similarly, for Histidine: H68 and I44 with *d*_*α*−*α*_ ~ 0.5 nm, the plots of 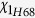 of H68 and 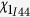 of I44 as functions of time (Fig. 1(b)), reveal anti-correlated behavior even at long times.

Now we proceed to quantify temporal correlations between these time series. We extract the TDCF between dihedral *θ* of residue *i* and *θ*′ of residue *j* in time interval *Δt* from equilibrated trajectory (see Supplementary Fig. S3). TDCF is denoted as 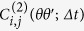, for a time interval *Δt* = *t*_2_ − *t*_1_ (see Methods, Eqs 1 and 2, *Note 2*). We compute TDCF from MD trajectory for three different sets of maximum time up to *t* = 500 ns, 950 ns and 1.05 μs of the simulated trajectory. Since the correlation functions are computed from the dihedral values at two time intervals over trajectory, averaging at larger time interval gets better with longer time trajectory. We show 
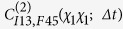
 (Fig. 1(c)) and 
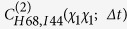
 (Fig. 1(d)) for three different cases. Both Fig. 1(c) and (d) show that data for trajectory up to *t* = 500 ns have differences with respect to larger time trajectories. However, data with trajectory up to *t* = 950 ns and 1.05 μs are comparable, indicating saturation in the temporal behavior of the TDCFs.

**Figure 2:**
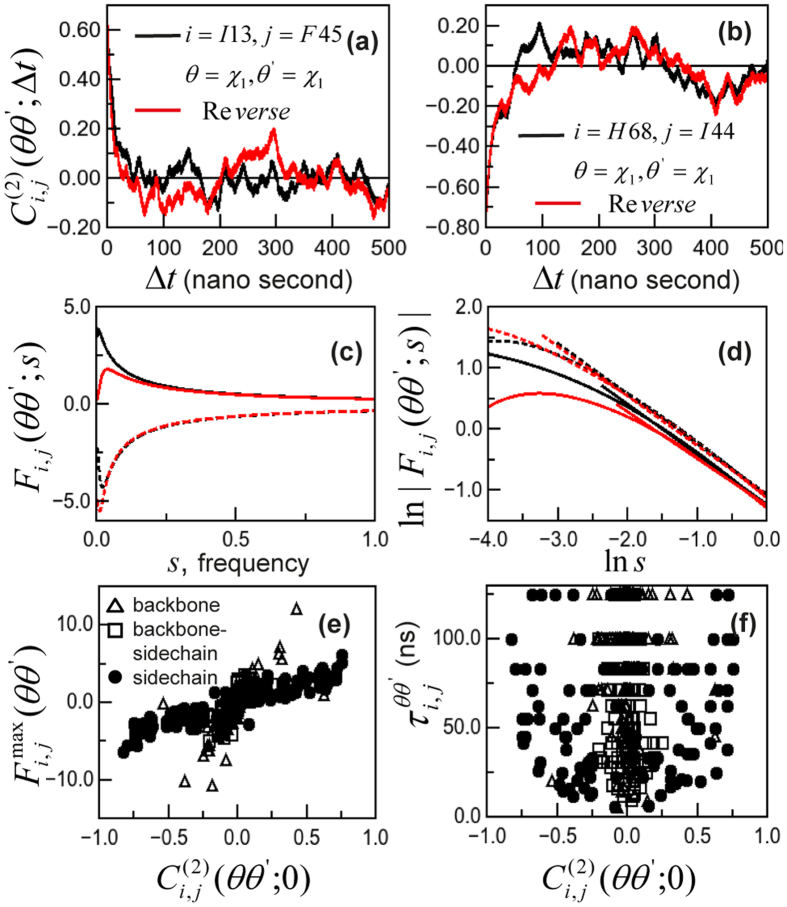
TDCFs between various dihedrals of ubiquitin, (Black: forward direction, red: reverse direction); (**a**) 
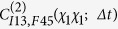
. (**b**) 
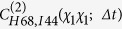
 as function of *Δt*. (**c**) Laplace Transform *F*_*i,j*_(*θθ*′; *s*) of correlation function, *F*_*I*13,*F*45_(*χ*_1_*χ*_1_; *s*) (solid line) and 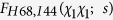 (dashed line) versus ***s*** plots. (**d**) ln |*F*_*I*13,*F*45_(*χ*_1_*χ*_1_; *s*)| versus ln *s* (solid line) and ln |*F*_*H*68,*I*44_(*χ*_1_*χ*_1_; *s*)| versus ln*s* (dashed line) plots showing algebraic tails. (**e**) Correlations plot between functionally important residues of ubiquitin; *F*_***i**,**j***_(*θθ*′; *s*) versus 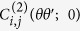. (**f**) 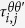 versus 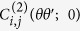 for similar residues. The symbols have the same meaning in (**e**) and (**f**).

We further report our analysis based on all the data for the longest trajectory, *t* = 1.05 μs. Figure 2(a) and (b) bring out further non-trivial aspect of the TDCFs, exhibited by several dihedral pairs, despite large separation between the residues. We show TDCFs for both forward and reverse direction, obtained by interchanging *i* and *j* and *θ* and *θ*′ for the longest trajectory. 
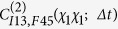
 (Fig. 2(a)) shows statistical Pearson Correlation, 
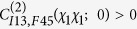
 at *Δt* = 0. This decays with increasing time interval. 
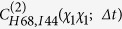
, (Fig. 2(b)) shows statistical Pearson anti-correlation, 
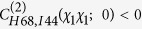
 at *Δt* = 0 following which it decays to zero for large *Δt*. We also observe in Fig. 2(a) and (b) that 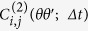 is different in forward and reverse directions, the decay time scales being different indicating asymmetry in TDCFs.

Figure 2(c) shows representative cases of Laplace Transform *F*_*i,j*_(*θθ*′; *s*) of 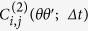, the correlation function (for additional cases see Supplementary Fig. S4). For small *s, F*_*I*13,*F*45_(*χ*_1_*χ*_1_; *s*) has a maximum where there is a statistical correlation (Fig. 2(a)), while *F*_*H*68,*I*44_(*χ*_1_*χ*_1_; *s*) < 0 and having a minimum in case of statistical anti-correlation (Fig. 2(b)). The asymmetry in *F*_*i,j*_(*θθ*′; *s*) under interchanges of *i* and *j* and *θ* and *θ*′ is evident from Fig. 2(c). The peak value of *F*_*i,j*_(*θθ*′; *s*) in *s*, 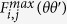 is a measure of the strength, and the inverse of peak position gives a characteristic time scale 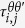 of correlation. These time scales (see Supplementary Table S1) are tens of nanoseconds, in the regime of rotational diffusion time much larger than atomic fluctuation time scales. The log-log plots in Fig. 2(d) show decaying tail (*s*^−κ^) with exponent *κ* for large *s.* The values of *κ* (see Supplementary Table S1) show quasi-universality (*κ* ~ 1.0).

Let us now examine the TDCFs for functionally relevant residues^9^ of ubiquitin. In recent simulation studies^9^ statistical Pearson correlations are observed between back-bone dihedrals of residue pairs (I13-F45, Threonine: T14-F45, Lysine: K6-F45 and I13-Leucine: L67, I13-Valine: V5-K6, K6-H68, H68-I44 and I44-F45) of ubiquitin, which belong to the binding surface patch of Ub. We plot 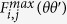 and 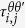 for all these residue pairs as functions of 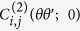 in Fig. 2(e) and (f) respectively. We observe that a strong correlation exists between peak values of TDCFs and 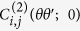. However, the timescales are not correlated to 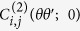. This is not surprising, for statistical correlation coefficients do not contain temporal information.

## Functional relevance

**Figure 3:**
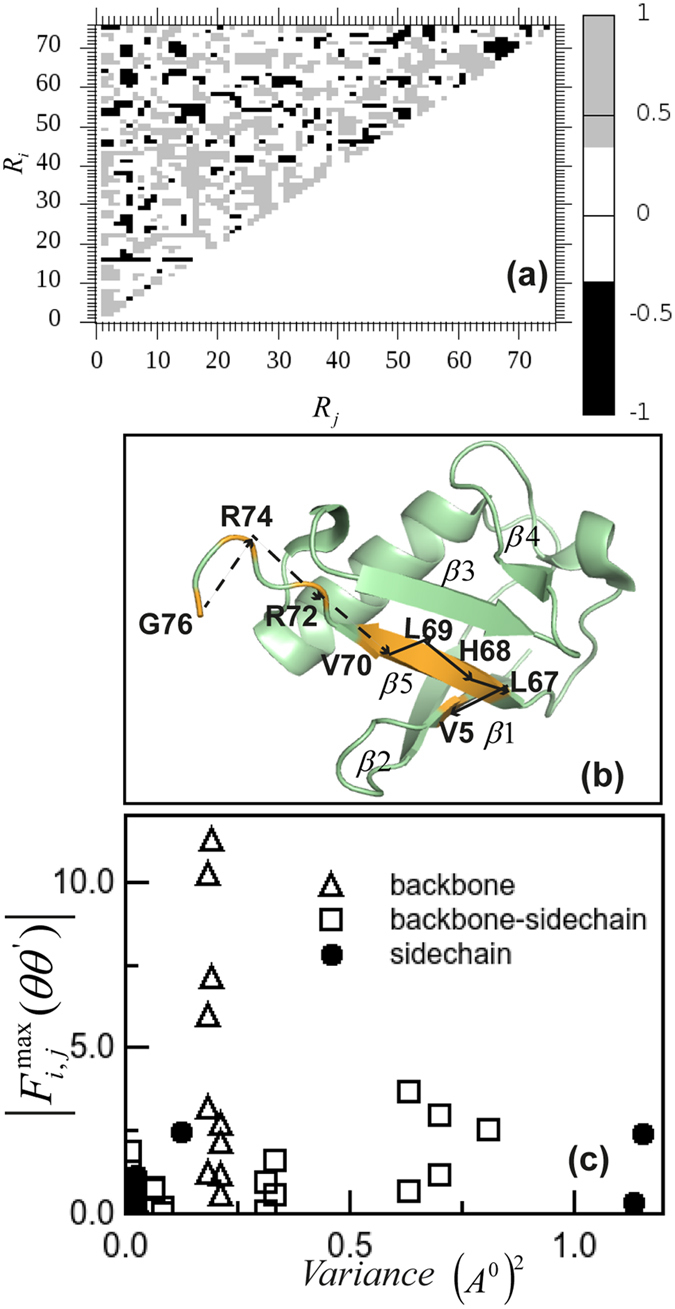
(**a**) TDCF map for any two residue pair in ubiquitin; Black represents downstream and Grey represents upstream TDCFs. (**b**) Residues belong to dynamically correlated path of ubiquitin. Solid line connects the residue pairs belong to β-sheets, dashed line connects the pairs belong to the loop region. (**c**) Correlation peak versus distance fluctuations of residue pairs belong to temporally correlated path.

Our analysis yields a detailed map of correlated residue pairs *R*_*i*_ and *R*_*j*_, as shown in Fig. 3(a). For a particular residue pair *R*_*i*_ and *R*_*j*_ we compute 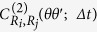 for every possible pairs of degrees of freedom (dof), like 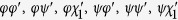, *χ*_1_*φ*′, *χ*_1_*ψ*′, 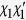. The dof pairs for which 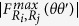 is maximum is considered to determine the direction of correlation in time domain, namely, if any perturbation at *R*_*i*_ affects *R*_*j*_ at a later time or vice versa. We generate a 76 × 76 matrix by noting 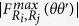 for all of the 76 residues of ubiquitin. By applying the condition of directionality we obtain the upper triangular matrix showing detailed TDCF map.

This map can be used to understand correlated path among the residues. Let us consider the terminal residue G76 which binds to AMP during Ub activation in ubiquitination. The dihedral *φ* of G76, *φ*_*G*76_ is correlated to R74 by *φ*_*R*74_, *ψ*_*R*74_ and *χ*_1*R*74_ both in forward and reverse direction. However, among all these correlated dihedrals 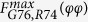 is the largest which we take as an indication that G76 is downstream correlated to R74 via dihedral *φ* of both the residues. Similarly G76 is downstream correlated to other set of residues, like L73, L67, Glutamine: Q62, Tyrosine: Y59, L56, R54, Aspartate: D52, K48, F45, L43, Q41, Q40, D39, Proline: P38, K33, I30, K27, V26, Glutamate: E24, I23, D21, T12, T7 and V5. Among all the downstream correlated residues to G76 we find that R74 is having the shortest *d*_*α*−*α*_, which is the mean distance between *C*_*α*_ atoms over the entire trajectory. Similarly, the closest downstream correlated residue to R74 is R72. In this way we construct the path of downstream correlated dihedrals of different residues, 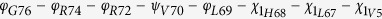, as shown in a snapshot of ubiquitin obtained from simulation (Fig. 3(b)). Among these temporally correlated residues G76, R74 and R72 belong to the C terminal loop region. The residues V70, L69, H68 and L67 belong to β5, while V5 belongs to β1 in β strands of the crystal structure^2^. Crystal structure of ubiquitin activation enzyme-E1 loaded with Ub molecules indicates^4^,^5^ that the hydrophobic surface patch of Ub including L8, I44, V70 and C terminal tail of Ub (G76, R74, R72) interact with the activation enzyme. The temporally correlated path with G76 contains many of the residues, like R74, R72 and V70. Besides, the slowest time scale in this path is that between G76 and R74, around 125 ns. This time scale is comparable to the rotational time scale of the enzyme which is about 90 ns obtained using the Stokes-Einstein^25^ equation. Thus the path obtained using TDCF analysis is functionally relevant.

In order to get mechanistic view of long distance correlations, we calculate variance of the distances between residues belonging to the temporally correlated path. We compute *var(d*_*α*−*α*_) which represents variance of *d*_*α*−*α*_. Similarly for backbone-side chain distances, we calculate *var(d*_*α*−*β*_), where *d*_*α*−*β*_ denotes distance between *C*_*α*_ and *C*_*β*_ atoms of the residue pair. For side chain dihedrals, we compute *var(d*_*β*−*β*_), the variance of distance between *C*_*β*_ atoms of the correlated pairs. We plot 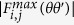 versus these variances in Fig. 3(c). We observe that the large correlation amplitudes are clustered near smaller values of the variance. This indicates that dihedral dynamical correlations are destroyed by large fluctuations.

We compute transfer entropy from mutual information^26^ between maximally correlated pairs of fluctuating degrees of freedom which constitute the functionally relevant path. We assign the directionality of entropy transfer between two residues by the larger magnitude of the transfer entropy for the correlated pairs in forward and reverse directions. For instance, in case of 
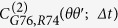
, *φ*_*G*76_ and *φ*_*R*74_ are the maximally correlated dof both in forward and reverse directions (see Methods, *Note 3*). We find that mutual transfer entropy −0.17 for *φ*_*R*74_ to *φ*_*G*76_ and that for the reverse direction is 0.55, indicating that the transfer of information takes place from *φ*_*G*76_ to *φ*_*R*74_ similar to experimental observations. We construct in similar way the direction of entropy transfer and time scales of the correlated dof over the path, as given in Supplementary Table S2. It is clear from the table that the directionality of path is not maintained between R74 and R72 where the entropy transfer takes place from R72 to R74. Moreover, the time scale of optimum mutual information is in sub-ns range, orders of magnitude shorter than biologically relevant time scales. Thus the TDCF describes the functionally relevant path in more reliably.

## Mathematical Model

We model the qualitative behaviors of the TDCF in terms of equations of motion (see Methods, Eqs 3 and 4, *Note 4* and for the details of calculations see Supplementary Note. S1) of two dihedrals *θ*_*i*_(*t*) and *θ*_*j*_(*t*) which are coupled to each other with strengths *α*′ and *β*′ respectively. Let the characteristic frequencies associated with them be *ω*_*i*_ and *ω*_*j*_. They perform motions in a solvent experiencing drags proportional to 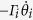 and 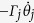 respectively^18^. We calculate the Laplace transformed correlation function from the equations of motion, 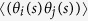 in frequency, *s* by averaging over initial conditions on the variables. We find that in *s* → 0 limit, 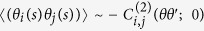 
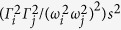
, 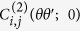 being the statistical correlation coefficient. There is thus maximum in the low *s* limit if the TDCF shows statistical correlation, 
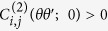
, while a minimum for statistically anti-correlated TDCF with 
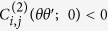
. These are qualitatively similar to low *s* behaviour of the simulated TDCFs. We get an algebraic tail for large *s*, 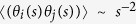 where the exponent is universal and independent of the parameters in the model. This universality is revealed by the simulated TDCF, albeit with exponent 1.0. The difference in the exponent may be due to simplicity of the model equations of motion where all effects are neglected except solvent drag and mutual coupling. Moreover, we find that so far as *α*′ ≠ *β*′, 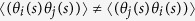 as seen in simulations.

Direct probe of dynamical correlation among the dihedrals is difficult due to limitation of probes. However, our analysis suggests an indirect way of probing the dynamical correlations. Our analysis shows that the residues, like R74, R72 and V70 lie in dynamically correlated path with G76 where ubiquitination initiates. We expect these residues to play an important role in the process which can be tested experimentally. R72 is experimentally known to give specificity to the ubiquitin activation enzyme-E1 binding^4^,^5^. The role of the other residues needs to be looked into.

To conclude we show with long molecular simulations and mathematical modeling that TDCFs explain the causal connection between binding sites in Ub in the biologically relevant temporal regime. More importantly, our studies indicate that non-linearties are not the primary deciding factor for causal connections between functional sites. Although the simulations are illustrated for ubiquitin activation enzyme-E1 binding to Ub, the generality of our mathematical analysis shows that qualitative features of TDCF can be extended to any microscopic degrees of freedom. On a wider perspective, two point cross-correlation functions between relevant microscopic variables may provide a correct description of bio-molecular function and the related kinetics in terms of underlying microscopic dynamics without invoking the nonlinear effects.

## Methods

### Note 1: Details of MD Simulation

We perform MD simulation using NAMD at 310 K and 1 atm pressure, following standard protocols for NPT ensemble. We use TIP3P water model, periodic boundary condition and CHARMM27^27^ force field with 1 femto-second time step. Electronutrality is maintained by adding proper number of mono-valent ions Na^+^ and Cl^−^. Long ranged electrostatic interaction is included by PME^28^ method. Energy minimization was done for first 10,000 steps and simulation was performed for 1.05 μs. Equilibration is ensured by RMSD plot over entire simulation time.

### Note 2: Details of computation of two-point correlation function (TDCF)

Dihedral angle is the intersecting angle between two adjacent planes for four consecutive atoms. In case of polypeptide backbone for *C*_*i*−1_ − *N*_*i*_ − *C*_*α,i*_ − *C*_*i*_, angle between *C*_*i*−1_ − *N*_*i*_ − *C*_*α,i*_ and *N*_*i*_ − *C*_*α,i*_ − *C*_*i*_ planes is known as dihedral φ. Similarly angle between *N*_*i*_ − *C*_*α,i*_ − *C*_*i*_ and *C*_*α,i*_ − *C*_*i*_ − *N*_*i*+1_ is dihedral ψ where *i* is residue index. Proteins also possess side-chain dihedrals among which *χ*_1_ defines the angle between *N*_*i*_ − *C*_*α,i*_ − *C*_*β,i*_ and *C*_*α,i*_ − *C*_*β,i*_ − *C*_*γ,i*_. Thus the dihedral angles can be computed from the specified atomic positions obtained from the simulated MD trajectory.

The computation of TDCF from the MD trajectory has been done as follows: The series of the conformations is ordered in time with a given choice of initial condition. For any given time-difference *Δt* = |*t*_2_ − *t*_1_|, we compute the product between fluctuations of dihedral *θ* of residue *i* and *θ*′ of residue *j* for the *l*–th observation for a given *Δt*,


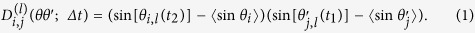


The angular bracket signifies ensemble average or mean of the respective quantity over simulation trajectory. The TDCF is given by 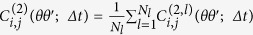, where


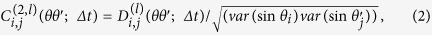


*var* denoting the variance of quantity within parenthesis. Here *N*_*l*_ is the number of observations corresponding to given *Δt*. For instance, for *N* number of observations, *N*_0_ = *N*. Similarly, *N*_1_ = *N* − 1 which correspond to the data set (*t*_1_ = 0, *t*_2_ = 2), … (*t*_1_ = *N* − 1, *t*_2_ = *N*).

The computation is done for sufficiently large *Δt* until 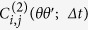 approaches zero. We compute numerically the Laplace Transform 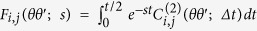.

### Note 3: Computation of transfer entropy

We use standard methodology for the computation of transfer entropy^15^. First we calculate the minimal embedding dimension (m)^26^ for two time series by the false nearest neighbors method^29^. Next we compute mutual information (MI) using the TISEAN package^26^ for fluctuations of pairs of dihedral angles for different residues belonging to the path. We identify the embedding time intervals (τ) for which MI is optimum^15^,^26^. We compute the transfer entropy^15^ between two degrees of freedom from the Shannon entropy and joint entropy using the MIToolbox^30^ for fluctuations of two time series using m and τ.

### Note 4: Over damped equations of motion in the long time limit

We have used equation of motion of over damped coupled classical harmonic oscillators.


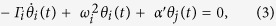



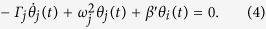


Using Laplace Transform of equations (3) and (4) we model behavior of TDCF in *s* domain. Details are in Supplementary Note S1.

## Additional Information

**How to cite this article**: Dutta, S. *et al*. Spatio-temporal coordination among functional residues in a protein. *Sci. Rep.* **7**, 40439; doi: 10.1038/srep40439 (2017).

**Publisher's note:** Springer Nature remains neutral with regard to jurisdictional claims in published maps and institutional affiliations.

## Supplementary Material

Supplementary Information
