# Supplementary material for: Spatio-temporal coordination among functional residues in protein
Source: Sci Rep. 2017 Jan 16;7:40439. doi: 10.1038/srep40439 (PMC5238388; doi:10.1038/srep40439)
Supplement: Supplementary Information [file srep40439-s1.pdf]

Supplementary Material:

Spatio-temporal coordination among functional residues in protein

Sutapa Dutta, [1] Mahua Ghosh, [1] and J. Chakrabarti[1, 2,*]

[1]Department of Chemical, Biological And Macro-Molecular Sciences, S. N. Bose National Centre for Basic Sciences, Sector III, Block JD, Salt Lake, Kolkata 700106, India

[2]Also at Unit of Nanoscience and Technology-II and The Thematic Unit of Excellence on Computational Materials Science, S. N. Bose National Centre for Basic Sciences, Sector III, Block JD, Salt Lake, Kolkata 700106, India

Corresponding Authors: Email: *jaydeb@bose.res.in

Supplementary Note S1:

Over damped equations of motion in the long time limit:

The over damped equations of motion in long time limit are:

$$-\Gamma_i\dot{\theta}_i(t) + \omega_i^2\theta_i(t) + \alpha'\theta_j(t) = 0 \qquad (1),$$

$$-\Gamma_j\dot{\theta}_j(t) + \omega_j^2\theta_j(t) + \beta'\theta_i(t) = 0 \qquad (2).$$

Using Laplace transforms of equations (1) and (2) we obtain,

$$-s\Gamma_i\theta_i(s) + \omega_i^2\theta_i(s) + \alpha'\theta_j(s) = -\Gamma_i\theta_i(0) \qquad (3),$$

$$-s\Gamma_j\theta_j(s) + \omega_j^2\theta_j(s) + \beta'\theta_i(s) = -\Gamma_j\theta_j(0) \qquad (4),$$

or writting equation (3) and (4) in matrix form:

$$\begin{pmatrix} \omega_i^2 - s\Gamma_i & \alpha' \\ \beta' & \omega_j^2 - s\Gamma_j \end{pmatrix} \begin{pmatrix} \theta_i(s) \\ \theta_j(s) \end{pmatrix} = -\begin{pmatrix} \Gamma_i\theta_i(0) \\ \Gamma_j\theta_j(0) \end{pmatrix} \qquad (5).$$

Denoting,

$A = \begin{pmatrix} \omega_i^2 - s\Gamma_i & \alpha' \\ \beta' & \omega_j^2 - s\Gamma_j \end{pmatrix}$ and $DetA = \begin{vmatrix} \omega_i^2 - s\Gamma_i & \alpha' \\ \beta' & \omega_j^2 - s\Gamma_j \end{vmatrix}$ we get,

$$\begin{pmatrix} \theta_i(s) \\ \theta_j(s) \end{pmatrix} = -\begin{pmatrix} \omega_i^2 - s\Gamma_i & \alpha' \\ \beta' & \omega_j^2 - s\Gamma_j \end{pmatrix}^{-1} \begin{pmatrix} \Gamma_i\theta_i(0) \\ \Gamma_j\theta_j(0) \end{pmatrix},$$

$$\begin{pmatrix} \theta_i(s) \\ \theta_j(s) \end{pmatrix} = -\frac{1}{DetA}\begin{pmatrix} \omega_j^2 - s\Gamma_j & -\alpha' \\ -\beta' & \omega_i^2 - s\Gamma_i \end{pmatrix} \begin{pmatrix} \Gamma_i\theta_i(0) \\ \Gamma_j\theta_j(0) \end{pmatrix}.$$

Equation (5) yields the following expressions:

$$\theta_i(s) = -\frac{1}{DetA}\left[\Gamma_i\theta_i(0)\left(\omega_j^2 - s\Gamma_j\right) - \alpha'\Gamma_j\theta_j(0)\right] \quad (6),$$

$\theta_j(s) = -\frac{1}{DetA}\left[\Gamma_j\theta_j(0)(\omega_i^2 - s\Gamma_i) - \beta'\Gamma_i\theta_i(0)\right]$ (7).

We construct the product between $\theta_i(s)$ and $\theta_j(s)$ is using equation (6) and (7) taking average over initial condition to get the TDCF,

$$< \left(\theta_i(s)\theta_j(s)\right) > = \frac{1}{(Det\ A)^2} \times [-\Gamma_i{}^2\left(\omega_j^2 - s\Gamma_j\right)\beta' < \theta_i(0)\theta_i(0) >$$

$$+ \alpha'\beta'\Gamma_i\Gamma_j < \theta_j(0)\theta_i(0) > + \left(\omega_j^2 - s\Gamma_j\right)(\omega_i^2 - s\Gamma_i)\Gamma_i\Gamma_j < \theta_i(0)\theta_j(0) >$$

$$- \alpha'\Gamma_j{}^2(\omega_i^2 - s\Gamma_i) < \theta_j(0)\theta_j(0) >] \qquad (8).$$

Here, $< \theta_i(0)\theta_i(0) > = C_{ii} = 1, < \theta_j(0)\theta_j(0) > = C_{jj} = 1$ and

$< \theta_i(0)\theta_j(0) > = C_{i,j}^{(2)}(\theta\theta'; 0) = < \theta_j(0)\theta_i(0) >$.

Introducing these in equation (8),

$$< \left(\theta_i(s)\theta_j(s)\right) > =$$

$$\frac{1}{(Det\ A)^2} \times [\Gamma_i{}^2\Gamma_j{}^2C_{i,j}^{(2)}(\theta\theta'; 0)s^2 + s\Gamma_i\Gamma_j\left[\left(\beta'\Gamma_i + \alpha'\Gamma_j\right) - C_{i,j}^{(2)}(\theta\theta'; 0)\left(\omega_j^2\Gamma_i + \omega_i^2\Gamma_j\right)\right] +$$

$$\left[\left(\Gamma_i\Gamma_jC_{i,j}^{(2)}(\theta\theta'; 0)\left(\omega_i^2\omega_j^2 + \alpha'\beta'\right)\right) - \left(\Gamma_i{}^2{\omega_j}^2\beta' + \alpha'\omega_i^2\Gamma_j{}^2\right)\right]] \qquad (9).$$

One can find from equation (9) that for large s, the leading term in numerator is s^2 and that in denominator is s^4. Thus $< \left(\theta_i(s)\theta_j(s)\right) > \sim s^{-2}$. Let us now consider $s \to 0$ limit.

Expanding the numerator and denominator in equation (9),

$$< \left(\theta_i(s)\theta_j(s)\right) > =$$

$$\frac{1}{\left(\omega_i^2\omega_j^2 - \alpha'\beta'\right)^2} \times (\Gamma_i{}^2\Gamma_j{}^2C_{i,j}^{(2)}(\theta\theta'; 0)s^2 + s\Gamma_i\Gamma_j\left[\left(\beta'\Gamma_i + \alpha'\Gamma_j\right) - C_{i,j}^{(2)}(\theta\theta'; 0)\left(\omega_j^2\Gamma_i\omega_i^2\Gamma_j\right)\right] +$$

$$\left[\left(\Gamma_i\Gamma_jC_{i,j}^{(2)}(\theta\theta'; 0)\left(\omega_i^2\omega_j^2 + \alpha'\beta'\right)\right) - \left(\Gamma_i{}^2{\omega_j}^2\beta' + \alpha'\omega_i^2\Gamma_j{}^2\right)\right]) \times$$

$$(1 + \frac{2s\left(\omega_j^2\Gamma_i + \omega_i^2\Gamma_j\right)}{\left(\omega_i^2\omega_j^2 - \alpha'\beta'\right)} - s^2\left[\frac{2\Gamma_i\Gamma_j}{\left(\omega_i^2\omega_j^2 - \alpha'\beta'\right)} + \frac{\left(\omega_j^2\Gamma_i + \omega_i^2\Gamma_j\right)^2}{\left(\omega_i^2\omega_j^2 - \alpha'\beta'\right)^2}\right]) \qquad (10).$$

From equation (10) we obtain coefficient of s^2,

$$\frac{\Gamma_i^2\Gamma_j^2 C_{i,j}^{(2)}(\theta\theta';0)}{\left(\omega_i^2\omega_j^2 - \alpha'\beta'\right)^2} + \left[\frac{2\left(\omega_j^2\Gamma_i + \omega_i^2\Gamma_j\right)\Gamma_i\Gamma_j}{\left(\omega_i^2\omega_j^2 - \alpha'\beta'\right)^3}\left\{\left(\beta'\Gamma_i + \alpha'\Gamma_j\right) - C_{i,j}^{(2)}(\theta\theta';0)\left(\omega_j^2\Gamma_i + \omega_i^2\Gamma_j\right)\right\}\right] -$$

$$\left[\left\{\frac{2\Gamma_i\Gamma_j}{\left(\omega_i^2\omega_j^2 - \alpha'\beta'\right)^3} + \frac{\left(\omega_j^2\Gamma_i + \omega_i^2\Gamma_j\right)^2}{\left(\omega_i^2\omega_j^2 - \alpha'\beta'\right)^4}\right\}\left\{\begin{array}{c}\left(\Gamma_i\Gamma_j C_{i,j}^{(2)}(\theta\theta';0)\left(\omega_i^2\omega_j^2 + \alpha'\beta'\right)\right) - \\ \left(\Gamma_i^2\omega_j^2\beta' + \alpha'\Gamma_j^2\omega_i^2\right)\end{array}\right\}\right].$$

Assuming $\omega_i^2\omega_j^2 \gg \alpha'\beta'$, consider the terms having highest order of $\Gamma_i\Gamma_j$ that is independent of $\alpha'\beta'$ and have $C_{i,j}^{(2)}(\theta\theta';0)$ dependence. In this limit the coefficient of s^2 is ~ $-C_{i,j}^{(2)}(\theta\theta';0)\frac{\Gamma_i^2\Gamma_j^2}{\left(\omega_i^2\omega_j^2\right)^2}$. Thus for statistical anticorrelation ($C_{i,j}^{(2)}(\theta\theta';0) < 0$) coefficient of s^2 becomes +ve quantity which implies minimum value of $F_{i,j}(\theta\theta';s)$ for low s. For initial correlation ($C_{i,j}^{(2)}(\theta\theta';0) > 0$) coefficients of s^2 remain –ve, implying a maximum of $F_{i,j}(\theta\theta';s)$ for small s.

Interchanging i and j and α' and β' in equation (1) and (2), it is easy to check that asymmetry in TDCF that $< \left(\theta_i(s)\theta_j(s)\right) > \neq < \left(\theta_j(s)\theta_i(s)\right) >$.

Supplementary Table:

Supplementary Table S1: Data for residue pairs belong to temporally correlated path and having functional importance.

Residue-pair	dof	Exponents(κ)	$\lvert F_{i,j}^{max}\ (\theta\theta')\rvert$	$\tau_{i.i}^{\theta\theta'}$(ns)
I13-V5	$\chi_1\ \chi_1$	0.7	2.84	45.5
T14-F45	$\chi_1\ \chi_1$	0.7	2.08	71.4
L69-H68	$\psi\ \chi_1$	0.7	0.26	125.0
H68-I44	$\chi_1\ \chi_1$	0.8	4.25	45.5
K6-F45	$\chi_1\ \chi_1$	0.8	2.82	50.0
I44-F45	$\chi_1\ \chi_1$	0.8	3.63	55.6
K6-H68	$\chi_1\ \chi_1$	0.8	5.27	83.3
I13-F45	$\chi_1\ \chi_1$	0.8	3.83	125.0
I13-L67	$\chi_1\ \chi_1$	0.8	2.68	125.0
R72-V70	$\varphi\ \psi$	0.9	2.74	100.0
G76-R74	$\varphi\ \varphi$	0.9	7.13	125.0
K6-T12	$\chi_1\ \varphi$	0.9	1.15	125.0
H68-L67	$\psi\ \chi_1$	1.0	0.31	41.7
R74-R72	$\varphi\ \varphi$	1.0	10.25	100.0
V70-L69	$\psi\ \varphi$	1.1	0.35	71.4
V5-K6	$\chi_1\ \varphi$	1.3	0.15	55.6
L67-V5	$\psi\ \chi_1$	1.3	2.49	71.4
K6-L67	$\chi_1\ \varphi$	1.6	0.25	83.3

Supplementary Table S2: Embedding dimension (m), optimal time interval (τ in ps) and transfer entropy for the residues belonging to temporally correlated path, both in forward and reverse direction.

Forward direction	(m,τ) of 1st residue	(m,τ) of 2nd residue	Transfer entropy in forward direction	Reverse direction	(m,τ) of 1st residue	(m,τ) of 2nd residue	Transfer entropy in reverse direction
$\varphi_{G76} - \varphi_{R74}$	5, 5	5, 18	0.55	$\varphi_{R74} - \varphi_{G76}$	5, 18	5, 5	-0.17
$\varphi_{R74} - \varphi_{R72}$	5, 18	5, 5	0.52	$\varphi_{R72} - \chi 1_{R74}$	5, 5	5, 20	0.76
$\varphi_{R72} - \psi_{V70}$	5, 5	5, 11	0.62	$\chi 1_{V70} - \chi 1_{R72}$	5, 20	5, 17	0.65
$\psi_{V70} - \varphi_{L69}$	5, 11	5, 4	-0.07	$\varphi_{L69} - \psi_{V70}$	5, 4	5, 11	0.53
$\varphi_{L69} - \chi 1_{H68}$	5, 4	5, 5	0.14	$\psi_{H68} - \chi 1_{L69}$	5, 2	5, 4	0.70
$\chi 1_{H68} - \chi 1_{L67}$	5, 5	5, 9	-0.35	$\chi 1_{L67} - \varphi_{H68}$	5, 9	5, 5	-0.27
$\chi 1_{L67} - \chi 1_{V5}$	5, 9	5, 10	0.66	$\varphi_{V5} - \chi 1_{L67}$	5, 3	5, 9	0.51

Supplementary Figure:

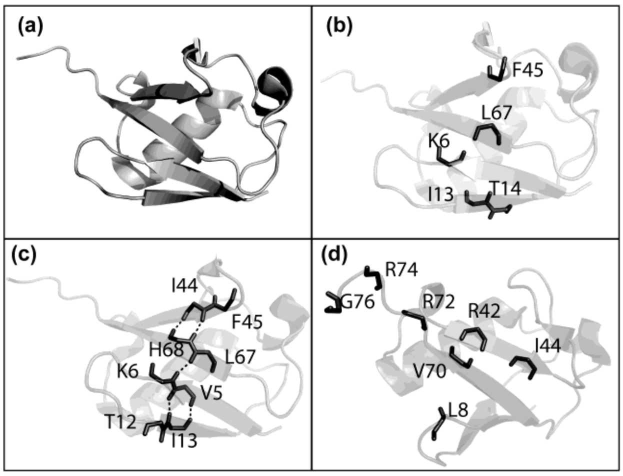

Supplementary Figure S1. Cartoon representation of ubiquitin: (a) Crystal structure of ubiquitin (PDB id: 1UBQ); (b) Correlated residues belonging to binding surface patch. (c) H-bonded residues. (d) Residues participating in binding with E1 enzyme, involved in ubiquitination. The dotted line in (c) connects the H-bonded pairs.

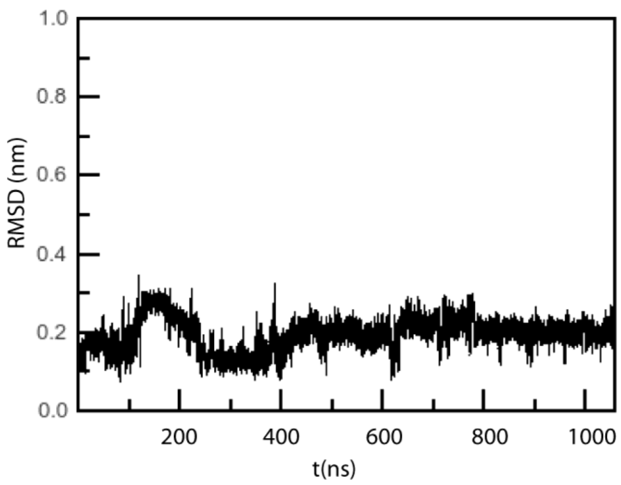

Supplementary Figure S2. RMSD plot of ubiquitin over 1.05 μs

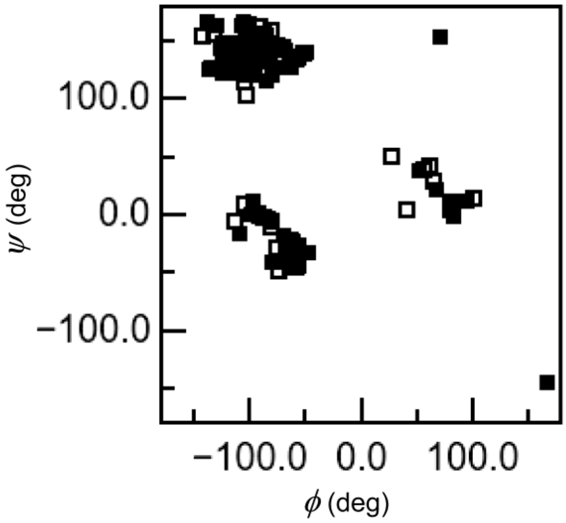

Supplementary Figure S3. $\psi - \varphi$ Correlation plot of residues in ubiquitin; filled rectangle represents the crystal structure and hollow rectangle shows the simulated average structure of ubiquitin obtained from the equilibrated trajectory.

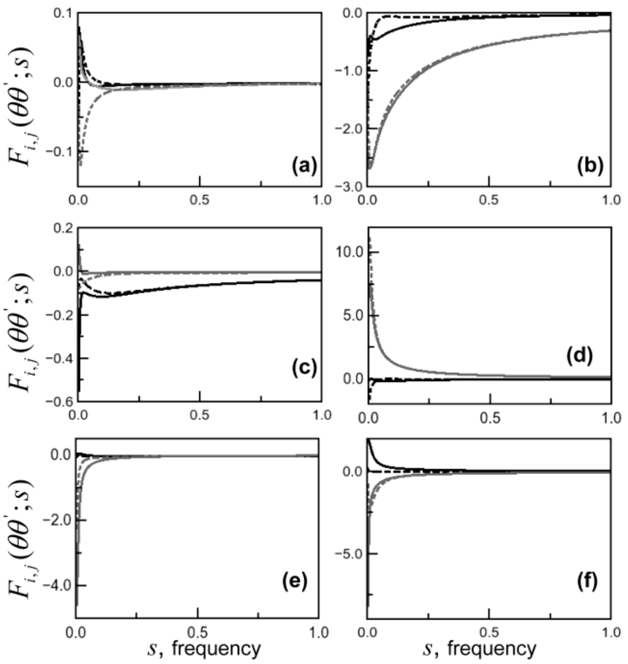

Supplementary Figure S4. Laplace transform of TDCFs between functionally important residues (Solid line: forward direction, dashed: reverse direction): (a) $F_{I13,L67}(\varphi\varphi;s)$ (black) and $F_{I13,L67}(\varphi\psi;s)$ (grey). (b) $F_{I13,L67}(\psi\chi_1;s)$ (black) and $F_{I13,L67}(\chi_1\chi_1;s)$ (grey). (c) $F_{I13,V5}(\psi\Phi;s)$ (black) and $F_{H68,I44}(\psi\varphi;s)$ (grey). (d) $F_{I13,V5}(\psi\chi_1;s)$ (black) and $F_{G76,R74}(\varphi\varphi;s)$ (grey). (e) $F_{V70,L8}(\varphi\psi;s)$ (black) and $F_{R42,R74}(\psi\psi;s)$ (grey). (f) $F_{V70,L8}(\varphi\chi_1;s)$ (black) and $F_{R42,R74}(\chi_1\psi;s)$ (grey).
